# Society Meetings

**Published:** 1902-09

**Authors:** 


### SOCIETY MEETINGS.

The Erie County Medical Association will hold its next regular quarterly meeting at the University Club, 295 Delaware Avenue, Buffalo, September 8, 1902. Drs. William H. Mansperger and James E. King will read papers, after which there will be social entertainment including a collation.

The Southern Surgical and Gynecological Association will hold its fifteenth annual meeting at Cincinnati, November 11 to 13, 1902, under the presidency of Dr. William E. B. Davis, of Birmingham. The secretary, Dr. William D. Haggard, Jr., of Nashville, in effect announces in his circular of invitation for papers, that in coming north of its original boundary lines the association will become national in its theory and practice—geographically and professionally. Hence it is to be anticipated the meeting announced will be the largest and most representative that has yet been held by the association. Those who desire to present papers should address Dr. Haggard at an early day.

---

The American Electro-therapeutic Association, will hold its twelfth annual meeting, September 2, 3 and 4, 1902, at Hotel Kaaterskill, Catskill Mountains, New York. The secretary is Dr. George E. Bill, of Harrisburg, Pa.

---

The American Röntgen Ray Society will hold its next annual meeting at Chicago, December 10 and 11, 1902, under the presidency of Dr. G. P. Girdwood, of Montreal. Dr. James B. Bullitt, of Kentucky, is the secretary, and Dr. John B. Murphy, of Chicago, is one of the members of the local committee of arrangements which is under the chairmanship of Dr. Ralph R. Campbell.

---

The New York State Homeopathic Medical Society will hold its semi-annual meeting at Utica, N. Y., September 16 and 17, 1902. Dr. DeWitt G. Wilcox, of Buffalo, is the secretary.

---

The American Dermatological Association will hold its twenty-sixth annual meeting at the Hotel Bellevue, Boston, September 18, 19 and 20, 1902, under the presidency of Dr. George Thomas Jackson, of New York. During the proceedings Dr. G. W. Wende, of Buffalo, will read a paper entitled, An unusual case of epidermolysis bullosa hereditaria.

---

The Mississippi Valley Medical Association will hold its 28th annual meeting at Kansas City, Mo., October 15-17, 1902, under the presidency of Dr. S. P. Collings, of Hot Springs, Ark. The preliminary program is out with a group of 55 papers already listed. The officers anticipate an unusually interesting meeting. An address in medicine will be delivered by Dr. Hugh T. Patrick, of Chicago, and an address in surgery, by Dr. Geo. W. Crile, of Cleveland. Further particulars can be had by addressing the secretary, Dr. Henry Enos Tuley, 111 Kentucky Street, Louisville.

---

The Medical Society of the Missouri Valley will hold its annual meeting, September 18, 1902, at Sioux City, Iowa. Among the papers to be read are the following: Vernal catarrh, especially in adults, H. Gifford, Omaha; Chronic spinal muscular atrophy, F. E. Coulter, Omaha; Surgical treatment of lesions of the stomach, J. E. Summers, Jr., Omaha. Dr. Richard C. Moore is president and Dr. Charles Wood Fassett is the secretary.

